# With Appreciation

**DOI:** 10.1002/jcsm.12367

**Published:** 2018-11-18

We thank the following individuals who served as manuscript reviewers for The Journal of Cachexia, Sarcopenia and Muscle (JCSM) in 2018.

We highly appreciate their efforts. Fair, conscientious and timely peer reviews contribute to the success of JCSM.

The Editors.

## 6 and more reviews

Andrea Bonetto

Denis Guttridge

Fabio Penna

Andrew Stewart Coats

## 3–5 reviews

Odessa Addison

Stephen Alway

Thiago Barbosa‐Silva

Daniel Bechet

Henri Bernardi

Richard Bohannon

Scott Bowen

Paola Costelli

Hans Degens

Jason Doles

Karyn Esser

Elisabetta Ferraro

Craig Goodman

Nicholas Greene

Michiyoshi Hatanaka

Satoshi Ikeda

Marco Invernizzi

Aminah Jatoi

Anu Kauppinen

Andy Khamoui

Michaël Laurent

Alessandro Laviano

Paul Lewandowski

Daniel Marks

Pablo Molina

Maurizio Muscaritoli

Carla Prado

Jeroen van Vugt

Yefei Wen

Jana Zschüntzsch

## 1–2 reviews

Eirik Aahlin

Sergio Adamo

Volker Adams

Camila Almeida

Koji Amano

Stefan Andreas

Hidenori Arai

Victor Arce

Atsushi Asakura

Asfar Azmi

Christine Baldwin

Vickie Baracos

Philipe Barreto

Elizabeth Barton

Oliver Bathe

Belinda Beck

Tarek Bekfani

Jose Berciano

David Berger

Mauricio Berriel Diaz

Bert Blaauw

Jeffrey Brault

Silvia Brunelli

Emma Bruns

Olivier Bruyere

Anton Bryantsev

Martin Burtscher

Jarrod Call

Elizabeth Carey

James Carson

Greg Cartee

Emanuele Cereda

Matteo Cesari

Ding‐Cheng Chan

Martin Chasen

Antonios Chatzigeorgiou

Philip Chilibeck

Jesper Frank Christensen

Paul Coen

Alexandru Corlateanu

Heather Cornnell

Maria Isabel Correia

Jeffrey Crawford

Jiujie Cui

Shalini Dalal

Srinivasan Dasarathy

Michael De Lisio

Boel De Paepe

Egidio Del Fabbro

Adolfo Díez‐Pérez

Francesco Dioguardi

Marcelo Dos Santos

Michael Drey

Esther Dupont‐Versteegden

Francois Durand

Debapriya Dutta

Nicole Ebner

Hirayuki Enomoto

William Evans

Alessandro Fanzani

Leonardo Ferreira

Katherine Flegal

Thomas Fleming

Brian Focht

Niels Focke

Martino V. Franchi

Allon Friedman

Christopher Fry

Nicholas Fuggle

Stephan Fuhrmann

Daniel Fuster

Silvana Gaetani

Jose Garcia

Giacomo Garibotto

Ghislaine Gayan‐Ramirez

Corinna Geisler

Duska Glavas

Maria Cristina Gonzalez

Brad Gordon

Andrea Graziani

E.M. Guinan

Cheng Guo

Anne Güttsches

Bishal Gyawali

Mark Hamrick

Jin Han

Justin Hardee

Michael Harris‐Love

Shinji Hatakeyama

Wei He

Jörg Heineke

David N Herndon

Stephan Herzig

Steven Heymsfield

Georgios Hillas

Susanna Hofmann

Michael Holick

Sabah Hussain

Robert Jackman

Courtney Jensen

Lee Jones

Keaton I Jones

Swati Joshi‐Barve

Andrew R. Judge

Boris Jung

Paweł Kabata

Kam Kalantar

Andreas Kavazis

Paul Kemp

Robert Kilgour

Won Kim

Serkan Kir

Katsuhiko Kohara

Aneta A. Koronowicz

Alexander Krannich

Franco Laghi

Barry Laird

Francesco Landi

Ramon C.J. Langen

Steen Larsen

Carl Lavie

Lorenz Lehmann

Christian Lehmann

Helmar Lehmann

Jennifer Le‐Rademacher

Vitor Lira

Medea Locquet

Jeremy Loenneke

Dawn Lowe

Tao Lu

Juliano Machado

Robert Mak

George Malietzis

Robert Mankowski

Giovanni Mantovani

Emanuele Marzetti

Loredana Mauro

Vera Mazurak

Huw McCarthy

Michela Meregaglia

Manuela Merli

Silvia Migliaccio

Maria del Pilar Milke Garcia

Ram Miller

Benjamin Miller

Martina Minnerop

William E. Mitch

Alessio Molfino

Hamid Moradi

Naoharu Mori

Pauline Morigny

John Morley

Marina Mourtzakis

Andrea Munsterberg

Antonio Musarò

Ryota Nakanishi

Enzo Nisoli

Kristina Norman

Julio Nunez

Antigone Oreopoulos

C.A. Ottenheijm

Andrea Pereira

Michele Petruzzelli

Rosanna Piccirillo

Andrew Poklepovic

Michael Polkey

Pascal Pomiès

Melissa Puppa

Zudin A. Puthucheary

Evelyn Ralston

Michael Reid

Jens Reimann

Sander Rensen

Erik Richter

Thomas Riediger

Michael Roberts

Eric Roeland

Maria Rohm

Vanina Romanello

Giuseppe Rosano

Rudolf Rudger

Kunihiro Sakuma

Stefano Schiaffino

Annemie Schols

Michael Schwarzer

Stylianos Scordilis

David Scott

Marília Seelaender

Thomas Seufferlein

Kunitoshi Shigeyasu

Ken Shirabe

Richard Skipworth

Will Smiles

Ashley Smuder

Tora Solheim

Leonardo Spataloa

Jochen Springer

Ivan Stankovic

Mick Steiner

Katharina Stoeck

Keiichi Sumida

Yu Sunakawa

Yoshihiko Tachi

Daniel Taillandier

Tetsuro Tamaki

Jeanette Thom

John Thyfault

Jose Trevino

Capucine Trollet

Madlen Uhlemann

Dilek Ural

Antonis Valachis

Gregory van der Kroft

Barbara van der Meij

David van Dijk

Konstantinos Volaklis

Stefano Volpato

Stephan von Haehling

Xiaonan Wang

Kevin Watt

Benjamn A. Weinberg

Christiane Weinrich

Kristin Werner

M.A. West

Sally Wheelwright

Grant Williams

Tracey Willis

Monte Willis

Simon Wing

Joachim Wiskemann

Carol Witczak

Robert Wolfe

Jean Woo

Junjie Xiao

YaNan Xing

Yosuke Yamada

Theresa Yeo

Seung Bae Yoon

N.E. Zanchi

Shankuan Zhu

Yao Zhu

Cheng‐le Zhuang

